# Diagnostic performance of fractional excretion of urea in the evaluation of critically ill patients with acute kidney injury: a multicenter cohort study

**DOI:** 10.1186/cc10327

**Published:** 2011-07-27

**Authors:** Michael Darmon, Francois Vincent, Jean Dellamonica, Frederique Schortgen, Frederic Gonzalez, Vincent Das, Fabrice Zeni, Laurent Brochard, Gilles Bernardin, Yves Cohen, Benoit Schlemmer

**Affiliations:** 1 Medical-Surgical Intensive Care Unit, Saint-Etienne University Hospital, and Jean Monnet University, Avenue Albert Raymond, F-42270 Saint-Etienne, France; 2 INSERM Unit 955, Paris-12 University, 51 Avenue du Marechal De Lattre de Tassigny, F-94010 Créteil, France; 3 Thrombosis Research Group, EA 3065, Saint-Etienne University Hospital, and Saint-Etienne Medical School, Avenue Albert Raymond, F-42270 Saint-Etienne, France; 4 Medical-Surgical Intensive Care Unit, Avicenne University Hospital, APHP, 125, rue de Stalingrad, F-93009 Bobigny, France; 5 Medical Intensive Care Unit, Archet University Hospital, Nice, France; and Nice University, UFR de Médecine, 151 Rte Saint Antoine Ginestiere, F-06202 Nice, France; 6 Medical Intensive Care Unit, AP-HP, Albert Chenevier-Henri Mondor University Hospital, and Paris-12 University, 51 Avenue du Marechal De Lattre de Tassigny, F-94010 Créteil, France; 7 Medical ICU, Saint-Louis University Hospital, APHP, Avenue Claude Vellefaux, F-75010 Paris, France; and UFR de Médecine, Paris-7 Paris-Diderot University, Avenue Claude Vellefaux, F-75010 Paris, France; 8 Medical-Surgical Intensive Care Unit, Hôpitaux Universitaires de Genève, 24, Micheli-du-Crest, CH-1211 Genève 14, Suisse; 9 Paris-13 University, 125, rue de Stalingrad, F-93009 Bobigny, France

**Keywords:** acute kidney failure, ICU, fractional excretion of sodium, acute tubular necrosis, diuretics, sensitivity and specificity

## Abstract

**Introduction:**

Several factors, including diuretic use and sepsis, interfere with the fractional excretion of sodium, which is used to distinguish transient from persistent acute kidney injury (AKI). These factors do not affect the fractional excretion of urea (FeUrea). However, there are conflicting data on the diagnostic accuracy of FeUrea.

**Methods:**

We conducted an observational, prospective, multicenter study at three ICUs in university hospitals. Unselected patients, except those with obstructive AKI, were admitted to the participating ICUs during a six-month period. Transient AKI was defined as AKI caused by renal hypoperfusion and reversal within three days. The results are reported as medians (interquartile ranges).

**Results:**

A total of 203 patients were included. According to our definitions, 67 had no AKI, 54 had transient AKI and 82 had persistent AKI. FeUrea was 39% (28 to 40) in the no-AKI group, 41% (29 to 54) in the transient AKI group and 32% (22 to 51) in the persistent AKI group (*P *= 0.12). FeUrea was of little help in distinguishing transient AKI from persistent AKI, with the area under the receiver operating characteristic curve being 0.59 (95% confidence interval, 0.49 to 0.70; *P *= 0.06). Sensitivity was 63% and specificity was 54% with a cutoff of 35%. In the subgroup of patients receiving diuretics, the results were similar.

**Conclusions:**

FeUrea may be of little help in distinguishing transient AKI from persistent AKI in critically ill patients, including those receiving diuretic therapy. Additional studies are needed to evaluate alternative markers or strategies to differentiate transient from persistent AKI.

## Introduction

Acute kidney injury (AKI) is common and associated with high mortality in critically ill patients [1-3]. The causes of AKI other than urinary tract obstruction are usually divided into two categories: prerenal causes, in which low renal perfusion leads to promptly reversible renal dysfunction, and intrinsic causes with renal tissue damage and persistent renal dysfunction. Although pathological studies are lacking, the leading cause of persistent AKI in critically ill patients is believed to be acute tubular necrosis (ATN) [4,5]. It is usually assumed that there is a continuum that leads from prerenal AKI to ATN [4-6]. Many publications in the fields of internal medicine, nephrology and critical care still advocate the use of urinary indices, such as the fractional excretion of sodium (FeNa), to differentiate transient from persistent AKI [4,5,7-10]. However, diuretic therapy or sepsis may affect these indices [11-13]. Since urea reabsorption occurs mainly at the proximal segment of the nephron and is unaffected by diuretic intake, the fractional excretion of urea (FeUrea) may be more reliable than FeNa [11,12,14]. Studies evaluating the performance of FeUrea have produced discordant results [11,12,14]. In addition, no study specifically designed to evaluate FeUrea in critically ill patients has been conducted. A recent review underlined the lack of evidence supporting the use of usual urinary indices in critically ill patients and in patients with sepsis [15]. However, distinguishing transient AKI from persistent AKI can help the clinician to choose the optimal treatment for critically ill patients.

Our primary objective in this study was to evaluate the performance of FeUrea as a tool for distinguishing transient from persistent AKI in a cohort of critically ill patients. The secondary objectives were to evaluate the performance of the usual urinary indices in these patients and to evaluate the performance of the usual urinary indices and FeUrea in the subgroup of patients receiving diuretics.

## Materials and methods

### Patients

The study was approved by the institutional review board of the French Society for Intensive Care Medicine (SRLF-CE-07-212), which waived the need for signed informed consent. Patients and their next of kin were informed, however, and none refused to participate. Three ICUs in university hospitals participated in the study between April and September 2008. Patients admitted to the participating ICUs were included, except those younger than 18 years of age, pregnant women, patients receiving dialysis for an underlying chronic kidney disease and patients with evidence of obstructive renal failure. Patients from whom urine could not be collected during the first six hours were excluded from this study.

### Protocol

Each patient was assessed during the first 12 hours following ICU admission. Plasma sodium, urea and creatinine levels were measured at ICU admission, and urine was collected over the next six hours.

### Definitions

AKI was defined according to the Acute Kidney Injury Network classification scheme [16] as a serum creatinine level increase of 26.4 μmol/L or more, a serum creatinine increase ≥ 150% from baseline or urine output < 0.5 mL/kg/hour for six hours or more. For patients whose baseline serum creatinine level was unknown, this variable was estimated using the Modification of Diet in Renal Disease (MDRD) formula [16,17].

Transient AKI was defined as AKI (of any stage) with a cause of renal hypoperfusion (that is, shock; dehydration; a medication interfering with renal perfusion, such as angiotensine-converting enzyme inhibitor; and so on) and recovery within three days. Recovery was defined as reversal of oliguria (in the absence of diuretics), and/or a 50% or greater decrease in serum creatinine [18], and/or return of serum creatinine to the baseline value (whether measured or estimated using the MDRD formula [16,17]). Persistent AKI was defined as renal dysfunction without recovery within three days. Oliguria was defined as urine output < 0.5 mL/kg/hour for six hours or more.

The FeNa percentage was calculated as ([urinary sodium/serum sodium]/[urinary creatinine/serum creatinine]) ×100. The FeUrea percentage was calculated as ([urinary urea/serum urea]/[urinary creatinine/serum creatinine]) ×100.

The Logistic Organ Dysfunction (LOD) score and the Simplified Acute Physiology Score version II (SAPS II) score were calculated at study inclusion [19,20], and the Knaus scale score was determined to evaluate chronic health status at ICU admission (A: no limitation of activity, B: moderate limitation, C: severe limitation, and D: bedridden or institutionalized) [21]. Sepsis was diagnosed using the criteria developed by the American College of Chest Physicians/Society of Critical Care Medicine consensus conference [22]. Individual organ failure was defined as a LOD score greater than 1 point for each system except the kidney [19].

### Statistical analysis

Patients remaining in the ICU for < 72 hours were secondarily excluded from the analysis, since they could not be classified as having transient or persistent AKI according to our definition. The results are reported as medians and interquartile ranges (IQRs), numbers and percentages or as means ± standard deviations (SD) to express the percentage changes. Categorical variables were compared using Fisher's exact test, and continuous variables were compared using the nonparametric Wilcoxon signed-rank test or the Mann-Whitney *U *test for pairwise comparisons. The Friedman test was used to compare continuous variables across the three groups.

To determine how well FeUrea distinguished transient from persistent AKI (our primary objective), we plotted the receiver-operating characteristic (ROC) curves of the proportion of true positives against the proportion of false positives, depending on the prediction rule used to classify patients as having persistent AKI. A 2 × 2 table was established to determine the sensitivity and specificity of FeUrea in diagnosing persistent AKI. Cutoff values, defined as threshold values that maximized the sum of sensitivity and specificity, were determined on the ROC curves. The positive and negative likelihood (LH) ratios were computed. The same strategy was used to assess our secondary objectives, namely, the performance of the usual urinary indices in these patients and the performance of the usual urinary indices and of FeUrea in the subgroup of patients receiving diuretics.

Last, to confirm the input of urinary indices to detect persistent AKI, we performed logistic regression analyses to identify variables significantly associated with persistent AKI measured by the estimated odds ratio (OR) with the 95% confidence interval (95% CI). Variables yielding *P *values < 0.20 in the bivariate analyses were entered into a backward stepwise logistic regression model in which persistent AKI was the variable of interest. The covariates were entered into the model with critical entry and removal *P *values of 0.2 and 0.1, respectively. Last, since the performance of FeUrea was the primary objective of this study, this variable was forced into the final model. Colinearity and interactions were tested. The Hosmer-Lemeshow test was used to check the goodness of fit of the logistic regression.

All tests were two-sided, and *P *values < 0.05 were considered statistically significant. Statistical tests were performed using the SAS version 6.12 software package (SAS Institute, Cary, NC, USA).

## Results

### Study population

During the study period, 203 patients with a median age of 61 years (46 to 73) were included. Their main characteristics are reported in Table 1. According to our definitions, 67 patients (33%) had no AKI, 54 patients (26.6%) had transient AKI and 82 patients (40.4%) had persistent AKI.

**Table 1:** Characteristics of patients without AKI, with transient AKI and with persistent AKI^a^

Demographics	No AKI (*n *= 67)	Transient AKI (*n *= 54)	Persistent AKI (*n *= 82)	*P *value^b^
Patient characteristics				
Male gender	34 (50.7%)	32 (59.3%)	56 (68.3%)	0.15
Age, years	50 (40 to 60)	71 (49 to 76)	66 (56 to 74)	< 0.0001
Weight, kg	68 (57 to 85)	75 (64 to 85)	80 (68 to 89)	0.006
Knaus score C or D [21]	21 (31.3%)	21 (38.9%)	40 (48.8%)	0.09
LOD score at ICU admission [19]	4 (2 to 7)	6 (5 to 9)	8 (5 to 9)	< 0.0001
SAPS II score at ICU admission [20]	35 (27 to 47)	50 (39 to 62)	52 (39 to 62)	< 0.0001
Risk factors for AKI				
Chronic heart failure	8 (11.9%)	14 (26.4%)	18 (22.0%)	0.15
Chronic kidney disease^c^	1 (1.5%)	3 (5.6%)	23 (28.0%)	< 0.0001
Sepsis	43 (64.2%)	33 (61.1%)	61 (74.4%)	0.12
Aminoglycosides	8 (11.9%)	9 (16.7%)	25 (30.5%)	0.2
Ionidated contrast agents	6 (9.0%)	3 (5.6%)	9 (11.0%)	0.55
Organ failure at ICU admission				
Medical condition	62 (92.5%)	51 (94.4%)	72 (87.8%)	0.36
Acute respiratory failure	54 (80.6%)	39 (72.2%)	61 (74.4%)	0.51
Coma	22 (32.8%)	24 (44.4%)	29 (35.4%)	0.34
Shock	22 (32.8%)	28 (51.9%)	43 (52.4%)	0.03
Treatments in the ICU				
Need for vasoactive drugs	20 (29.9%)	23 (42.6%)	43 (52.4%)	0.02
Mechanical ventilation	43 (64.2%)	34 (63.0%)	52 (63.4%)	0.99
Noninvasive mechanical ventilation	21 (31.3%)	14 (25.9%)	21 (25.6%)	0.73
Renal replacement therapy	4 (6.0%)	0	41 (50.0%)	<0.0001
Diuretics (at admission)	17 (25.4%)	18 (33.3%)	32 (39.0%)	0.21
Renal function at admission				
Diuresis, mL/kg/hour^d^	0.69 (0.59 to 0.99)	0.45 (0.32 to 1.11)	0.40 (0.21 to 0.72)	<0.0001
Plasma urea, mmol/L	5.4 (3.4 to 7.9)	13.1 (6.8 to 17.3)	17.4 (10.9 to 25.1)	<0.0001
Serum creatinine, μmol/L	68 (59 to 78)	124 (98 to 164)	220 (138 to 360)	<0.0001
Urinary indices				
Urine Na^+^/urine K^+^	1.8 (0.35 to 1.75)	1.0 (0.4 to 1.8)	1.3 (0.5 to 2.4)	0.01
FeNa, %	0.5 (0.3 to 1.3)	0.5 (0.2 to 1.3)	0.8 (0.4 to 4.0)	0.004
FeUrea, %	39 (28 to 40)	41 (29 to 54)	32 (22 to 51)	0.12
U/P urea	30 (19 to 39)	16 (9 to 25)	7 (4 to 14)	<0.0001
U/P creatinine	83 (52 to 127)	47 (25 to 76)	30 (11 to 58)	<0.0001
Outcomes				
ICU mortality	7 (11.7%)	13 (25%)	37 (48.1%)	0.0002
Hospital mortality	14 (20.9%)	15 (27.8%)	42 (51.2%)	0.0003

^a^AKI: acute kidney injury; LOD: Logistic Organ Dysfunction score, which can range from 0 to 22; SAPS II: Simplified Acute Physiology Score version II; FeNa, fractional excretion of sodium ([urine sodium/serum sodium]/[urine creatinine/serum creatinine]) ×100; FeUrea, fractional excretion of urea ([urine urea/serum urea]/[urine creatinine/serum creatinine]) ×100; U/P urea, urine urea/plasma urea; U/P creatinine, urine creatinine/serum creatinine. Data are medians (IQR) or number of patients (%). ^b^*P *values represent comparisons across the three patient groups. ^c^Chronic renal failure was defined as creatinine clearance < 60 mL/minute before ICU admission. ^d^Diuresis represents diuresis per kilogram and per hour during the first six hours following inclusion

At ICU admission, the median SAPS II score was 46 (34 to 60) and the median LOD score was 6 (4 to 9). Most patients were admitted for medical conditions (91.1%). The main risk factors for AKI were sepsis (67.5%), aminoglycoside therapy (20.7%), chronic heart failure (19.8%), chronic kidney disease (16.3%) and exposure to iodinated contrast agents (8.9%).

At the time of the study, no patient was being treated with renal replacement therapy (RRT). Forty-five patients required RRT during their ICU stay, usually during the first three days in the ICU (41 of 45 patients). Each of the patients requiring RRT during the first three days in the ICU had persistent AKI, whereas the remaining four patients had no AKI at ICU admission and required RRT later during their ICU stay.

### Diagnostic performance of FeUrea

**Figure 1:**
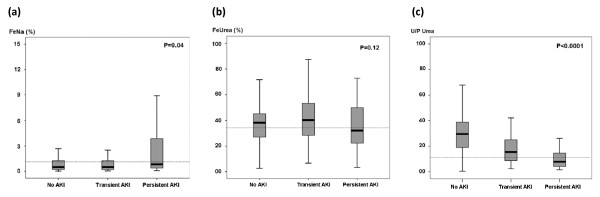
**(a) Boxplot of the fractional excretion of sodium (FeNa) in the overall study population according to renal function**. The dotted line represents FeNa of 1% (*P *= 0.04). **(b) **Boxplot of the fractional excretion of urea (FeUrea) in the overall study population according to renal function. The dotted line represents FeUrea of 35% (*P *= 0.12). **(c) **Boxplot of the urine/plasma (U/P) urea ratio in the overall study population according to renal function. The dotted line represents a U/P urea ratio of 10 (*P *< 0.0001).

Median FeUrea was 37% (26 to 49) overall, 39% (28 to 40) in patients without AKI, 41% (29 to 54) in patients with transient AKI and 32% (22 to 51) in patients with persistent AKI (*P *= 0.12). Figures 1a, b and 1c show the distributions of FeNa, FeUrea and urine/plasma (U/P) urea ratios, respectively, in each group.

**Figure 2:**
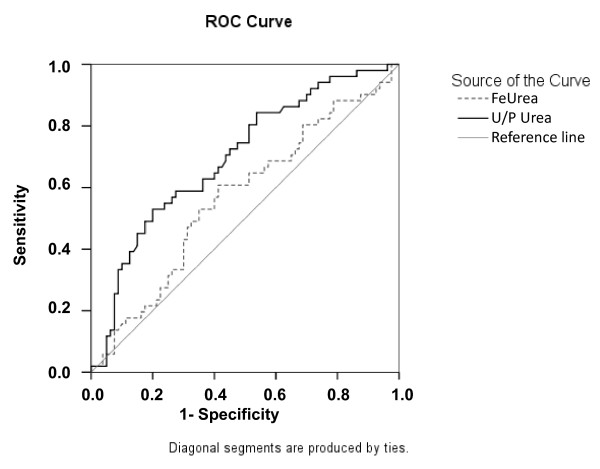
**Receiver-operating characteristic (ROC) curve depicting the ability of the fractional excretion of urea (FeUrea) and urine/plasma (U/P) urea ratio to detect persistent AKI in the subgroup of patients with AKI**. The ROC curve shows the relationship between the proportion of true positives (Sensitivity) and the proportion of false positives (1-Specificity) with various FeUrea and U/P urea ratio cutoffs. Diagonal segments are produced by ties. The area under the ROC curve is 0.59 (95% confidence interval, 0.49 to 0.70; *P *= 0.06) for FeUrea. The area under the ROC curve is 0.71 (95% confidence interval, 0.62 to 0.80; *P *= 0.04) for U/P urea ratio.

The area under the ROC curve was 0.59 (95% CI 0.49 to 0.70; *P *= 0.06) (Figure 2). At the usual cutoff (35%), FeUrea predicted persistent AKI with 63% sensitivity and 54% specificity (Table 2), yielding a positive LH of 1.37 and a negative LH of 0.68. In the study population, the optimal cutoff was 37%. However, the performance of FeUrea at this cutoff was poor (66% sensitivity and 53% specificity) (Table 2).

**Table 2:** Performance of usual urinary markers for detecting patients with persistent AKI among patients with AKI, with the usual and optimal (*) cutoff values^a^

Patient groups	FeNa > 1%	FeNa* > 0.58%	FeUrea < 35%	FeUrea* < 37%	U/P urea < 10	U/P urea* < 12	U/P creat < 20	U/P creat* < 12
All patients with AKI (*n *= 136; persistent AKI prevalence = 60.3%)								
Sensitivity (%)	0.48	0.63	0.63	0.66	0.74	0.66	0.79	0.59
Specificity (%)	0.7	0.61	0.54	0.53	0.57	0.66	0.39	0.59
Positive predictive value	0.71	0.71	0.67	0.68	0.72	0.74	0.66	0.68
Negative predictive value	0.47	0.47	0.47	0.51	0.59	0.56	0.55	0.48
Positive likelihood ratio	1.6	1.61	1.37	1.4	1.72	1.94	1.3	1.44
Negative likelihood ratio	0.74	0.61	0.68	0.64	0.45	0.52	0.54	0.69
Younden's index	0.18	0.24	0.17	0.19	0.31	0.32	0.18	0.18
ROC AUC	0.62 (0.52 to 0.72)		0.59 (0.49 to 0.70)		0.71 (0.62 to 0.80)		0.62 (0.53 to 0.72)	
Patients taking diuretics (*n *= 50; persistent AKI prevalence = 64%)								
Sensitivity (%)	0.75	0.62	0.61	0.61	0.72	0.61	0.78	0.89
Specificity (%)	0.56	0.56	0.47	0.59	0.69	0.75	0.5	0.38
Positive predictive value	0.71	0.71	0.67	0.68	0.81	0.81	0.73	0.72
Negative predictive value	0.47	0.47	0.49	0.51	0.58	0.52	0.56	0.66
Positive likelihood ratio	1.7	1.41	1.15	1.49	2.32	2.44	1.56	1.44
Negative likelihood ratio	0.47	0.68	0.83	0.66	0.41	0.52	0.44	0.29
Younden's index	0.31	0.18	0.08	0.2	0.41	0.36	0.28	0.27
ROC AUC	0.69 (0.54 to 0.81)		0.58 (0.41 to 0.75)		0.82 (0.70 to 0.94)		0.71 (0.56 to 0.86)	
Patients with sepsis (*n *= 94; persistent AKI prevalence = 65%)								
Sensitivity (%)	0.5	0.65	0.63	0.63	0.8	0.67	0.87	0.93
Specificity (%)	0.86	0.56	0.52	0.57	0.63	0.63	0.42	0.37
Positive predictive value	0.87	0.73	0.71	0.73	0.8	0.63	0.74	0.73
Negative predictive value	0.48	0.46	0.43	0.45	0.63	0.51	0.63	0.74
Positive likelihood ratio	3.57	1.48	1.31	1.47	2.1	1.81	1.5	1.48
Negative likelihood ratio	0.58	0.63	0.71	0.65	0.32	0.52	0.31	0.19
Younden's index	0.36	0.19	0.15	0.2	0.43	0.3	0.29	0.3
ROC AUC	0.67 (0.56 to 0.79)		0.56 (0.43 to 0.68)		0.71 (0.60 to 0.82)		0.65 (0.53 to 0.77)	

^a^AKI: acute kidney injury; ROC: receiver operating characteristic; AUC: area under the curve.

### Diagnostic performance of other urinary indices

The performance characteristics of classical urinary indices for detecting persistent AKI are reported in Table 2, with the usual and optimal cutoffs in the study population. Performance was best for the U/P urea ratio (ROC curve area under the curve (AUC) 0.71 (0.62 to 0.80)) (Figure 2). A U/P urea ratio < 12 had 66% sensitivity and 66% specificity for persistent AKI (positive LH, 1.94; negative LH, 0.52). When entered into a regression logistic model, none of these urinary indices were independently associated with persistent AKI. Three variables were found to be associated with persistent AKI: chronic kidney disease (OR 11.89, 95% CI 2.52 to 56.24; *P *= 0.02), need for vasopressors at ICU admission (OR 2.60, 95% CI 1.15 to 5.91) and oliguria at ICU admission (OR 2.50, 95% CI 1.11 to 5.63). The model had good calibration (goodness of fit *P *= 0.88). FeUrea was then forced into the final model and was not selected.

### Diagnostic performance of urinary indices in patients undergoing diuretic therapy

Overall, 67 patients (33%) received diuretics before or at ICU admission. Among them, 17 had no AKI (25.4% of patients without AKI), 18 had transient AKI (33.3% of patients with transient AKI) and 32 had persistent AKI (39% of patients with persistent AKI). The performance characteristics of urinary indices in patients undergoing diuretic therapy are reported in Table 2. As with the overall population, the performance of FeUrea in this patient subgroup was poor (ROC curve AUC 0.58 (0.41 to 0.75)). The U/P urea ratio performed satisfactorily in differentiating transient from persistent AKI (ROC curve AUC 0.82 (0.70 to 0.94)). With a U/P urea ratio cutoff of 10, sensitivity was 72%, specificity was 69%, positive LH was 2.32 and negative LH was 0.41.

### Diagnostic performance of urinary indices in patients with sepsis at ICU admission

Overall, 137 patients (67%) had sepsis at ICU admission. Among them, 43 had no AKI (64.2% of patients without AKI), 33 had transient AKI (61.1% of patients with transient AKI) and 61 had persistent AKI (74.4% of patients with persistent AKI). The performance characteristics of urinary indices in patients with sepsis are reported in Table 2. As with the overall population, the performance of FeUrea in this patient subgroup was poor (ROC curve AUC 0.56 (0.43 to 0.68)). The performance of other urinary indices was similar to that in the overall patient population.

## Discussion

In critically ill patients, FeUrea was not helpful in differentiating transient AKI from persistent AKI. Both in the overall population and in the subgroup of patients receiving diuretics, FeUrea performed less well than FeNa or the U/P urea ratio.

There is little scientific evidence to support the use of FeUrea. Only three studies have evaluated the accuracy of FeUrea in distinguishing transient from persistent AKI [11,12,14]. Their results are conflicting. In one study, FeUrea was 90% sensitive and 96% specific in differentiating transient from persistent AKI when a cutoff of 35% was used [11]. Conversely, another study found very poor diagnostic accuracy of FeUrea [12]. Several factors may explain these discordant results. First, these studies were single-center cohort studies and included only patients who were referred to nephrologists [11,12]. In addition, the study populations were poorly described but include both critically ill patients and patients in wards. Therefore, selection bias and differences between the institutions and study populations may explain the discrepancies [11,12]. Furthermore, FeUrea reflects the ratio of urea clearance over creatinine clearance ratio. Variations in creatinine clearance may therefore modify FeUrea. In the study that found good performance of FeUrea [11,12], wide differences in creatinine clearance can be suspected between patients with transient AKI and those with persistent AKI: serum creatinine levels were 140 ± 22 μmol/L and 520 ± 22 μmol/L (means +/- SD) in these two groups, respectively.

Interestingly, the performance of urinary indices in our study was poor. Several factors may explain this finding. First, although many publications have advocated the use of urinary biochemistry indices to differentiate transient from persistent AKI, these indices have not been extensively studied in critically ill patients or in patients with sepsis [4,5,23]. The few published studies have had several limitations: most of them were single-center case series or retrospective studies, the definition of AKI varied across the studies and the definition of transient AKI also varied, being subjective in most instances [24-30]. Several of these studies included patients who did not have critical illnesses [11,12,28]. In addition, we chose a definition allowing for a distinction between transient and persistent AKI. Our study was therefore not designed to evaluate the interest of these indices in distinguishing prerenal and intrinsic AKI. This point may partly explain the poor performance of the urinary indices in our study. Last, most of the studied patients had sepsis or shock at ICU admission. This condition is frequently associated with renal handling of sodium or water independently of an underlying AKI. This may also explain the poor performance of these indices in the studied population. Nevertheless, taking these factors into account, and although the usual urinary indices were able to differentiate transient from persistent AKI, the accuracy of the indices was poor and none of them were independently associated with the diagnosis of persistent AKI, indicating a need to identify other biomarkers.

Our study has several limitations. First, our definition of transient AKI was mainly based on renal function recovery. Indeed, an accurate definition of prerenal AKI would have required a highly subjective definition based on clinical histories, physical examinations and physicians' judgments [11,15]. In addition, AKI is mainly due to sepsis in critically ill patients, and in this setting there is frequently a continuum between volume depletion and persistent kidney injuries rather than two distinct entities, with the two mechanisms being frequently associated. Therefore, we chose a definition that relies only on an objective criterion. This point needs to be taken into account to interpret our findings. In the same way, the course of kidney function may have been modified by factors following study inclusion. However, in the ICU setting, predicting which patients will have persistent AKI may help to optimize treatment, such as by promptly restoring renal perfusion, limiting fluids or starting RRT. Our definition was highly sensitive for detecting patients with transient AKI (none of the patients in this group required RRT) but lacked specificity, since only 50% of patients in the persistent AKI group required RRT. Additional studies may help to determine the definition that best matches the need for RRT.

Second, although renal function was assessed within a few hours after ICU admission, the time course of the urinary indices was not evaluated. FeNa is known to vary during the first 12 to 24 hours in critically ill patients [13,30]. Few data are available on the time course of other urinary indices [13]. Any variations might explain the poor performance of FeNa or the other urinary indices. Therefore, these variations need to be investigated to determine the optimal time for renal assessment in critically ill patients.

Last, few of our patients received diuretics. The poor performance of urinary indices in this subgroup may therefore be related to low statistical power.

## Conclusions

In summary, we found that FeUrea and the usual urinary indices performed poorly in separating transient from persistent AKI in an unselected population of critically ill patients. Additional studies are needed to evaluate alternative markers of renal injury or strategies for differentiating transient from persistent AKI.

## Key messages

• FeUrea performed poorly in separating transient from persistent AKI in critically ill patients.

• Although the usual urinary indices (FeNa, U/P urea ratio or U/P creatinine ratio) are able to differentiate transient from persistent AKI, their accuracy remains poor in this setting.

• The high incidence of situations that may induce renal handling of water or sodium (that is, sepsis or shock) may explain the poor performance of urinary indices in this setting.

• Additional studies are needed to evaluate alternative markers of renal injury or strategies for differentiating transient from persistent AKI.

## Abbreviations

AKI: acute kidney injury; AUC: area under the curve; CI: confidence interval; FeNa: fractional excretion of sodium; FeUrea: fractional excretion of urea; MV: mechanical ventilation; OR: odds ratio; ROC: receiving operator characteristic; U/P creat: urine/serum creatinine ratio; U/P urea: urine/plasma urea ratio.

## Competing interests

The authors declare that they have no competing interests.

## Authors' contributions

MD had full access to all of the data in the study and takes responsibility for the integrity of the data and the accuracy of the data analysis. MD, FV and FS were responsible for the study concept and design. MD, FV, JD, FG and VD were responsible for the acquisition of data. MD, FV, FS, LB, GB, YC and BS analyzed and interpreted the data. MD and FV drafted the manuscript. *Critical revision of the manuscript for important intellectual content: *MD, FV, JD, FS, FG, VD, FZ, LB, GB, YC and BS critically revised the manuscript for important intellectual content. MD carried out the statistical analysis. All authors approved the final version of the manuscript.
